# Expression of integrin-linked kinase in the murine lens is consistent with its role in epithelial-mesenchymal transition of lens epithelial cells in vitro

**Published:** 2007-05-14

**Authors:** Matt S. Weaver, Natalie Toida, E. Helene Sage

**Affiliations:** 1 Hope Heart Program, Benaroya Research Institute at Virginia Mason, Seattle WA; 2 Department of Biological Structure, University of Washington, Seattle, WA

## Abstract

**Purpose:**

To evaluate the expression and location of integrin-linked kinase (ILK) within the mouse lens and to characterize the role of this protein during mouse lens epithelial cells (LEC) differentiation in vitro.

**Methods:**

Transcription levels of ILK mRNA were determined by RT-PCR in cultured cells and lens tissue. ILK protein was detected by immunoblotting, immunocytochemistry, immunohistochemistry, and immunoprecipitation. A role for ILK in the outgrowth of LEC from dissected mouse lens explants was determined by the use of ILK short interfering RNA (siRNA). Affinity-purified polyclonal anti-recombinant human ILK IgG was prepared and characterized for these experiments. A comparison of several anti-ILK antibodies was performed by immunoblotting, immunoprecipitation, and ELISA.

**Results:**

ILK was transcribed in LEC and lens fiber cells in vivo. ILK protein was expressed in the differentiating LEC at the equatorial region of the lens and, to a lesser extent, within the cortical and nuclear fiber cells. LEC in vitro produced copious ILK, which exhibited a filamentous pattern throughout the cytoplasm. The expression of ILK was increased during epithelial-mesenchymal-transition (EMT) of LEC from lens explants, whereas inhibition of ILK by siRNA delayed expression of the EMT markers smooth muscle α-actin and fibronectin.

**Conclusions:**

Analysis of ILK expression, localization, and activity in the mouse lens and cultured LEC is substantially facilitated by the generation of a multi-functional, polyclonal, affinity-purified anti-ILK antibody. Expressed in most tissues and cells lines, ILK is unexpectedly restricted to the equatorial LEC and differentiated fiber cells of the mouse lens. The occurrence of ILK expression with LEC differentiation is consistent with the positive regulatory function of ILK, which is revealed in a model of EMT in vitro. This is the first study to show the expression of ILK in the lens and its unique distribution pattern within cultured lens epithelia.

## Introduction

Integrin-linked kinase (ILK) is a serine-threonine kinase that binds to the cytoplasmic tails of β_1_- and β_3_-integrins (reviewed in [1]). It acts as an intermediate signaling protein during apoptosis/stress induction [2,3], differentiation [4,5], proliferation [6,7], and cellular interaction with the extracellular matrix (ECM) [8,9]. ILK has been shown to act downstream and independently of the phosphatidylinositol-3-kinase (PI3K) pathway to phosphorylate target proteins such as β_1_/β_3_-integrins, protein kinase B (Akt), and glycogen synthase kinase-3β (GSK-3β) [1]. Although significant publications have appeared describing the role of ILK in many tissues, in cancer biology (reviewed in [10]), and in several developmental systems, few studies have been conducted in the mammalian eye. It has been speculated that ILK is important in the lens because the motility, differentiation, ECM interaction, and survival of lens epithelia are required for lens development and function. It has also been suggested that, subsequent to cataract surgery, ILK could play a role in the requisite epithelial-mesenchymal-transition (EMT) of LEC, which contributes to the development of posterior capsular opacification (PCO) [11]. Consistent with this proposal, ILK has been identified as a regulator of EMT progression in several epithelia, e.g., renal and ovarian [12-14].

The majority of work on ILK has been performed with several commercially-available antibodies; the most commonly-used reagents are a mouse monoclonal antibody and rabbit polyclonal antibodies (Upstate Signaling, Lake Placid, NY). These antibodies appear to recognize alternate forms of ILK of 50 kDa and 60 kDa on immunoblots of cellular lysates. Despite these differences, the polyclonal antibody has been used for immunoprecipitation and subsequent ILK activity assays in the majority of recent studies. Neither of the antibodies has been used to show localization of ILK by staining of lens tissue.

To characterize ILK within the lens and its role in LEC EMT, we developed an affinity-purified, polyclonal antibody which recognizes both human and murine ILK by immunoprecipitation, immunohistochemistry, immunocytochemistry, and immunoblotting. With this antibody (R3B1) we determined the expression levels and localization of ILK within the murine lens. Furthermore, using ILK-targeting short interfering RNA (siRNA), we have shown that ILK is an important factor in the EMT of murine LEC, grown from lens explants.

## Methods

### Animals

All experiments were conducted in accordance with the ARVO Statement for the Use of Animals in Ophthalmic and Vision Research, and were carried out with the written permission of the relevant local institutional authorities.

### Antibodies

For immunoblotting, immunoprecipitation, and staining procedures, the following antibodies were used: monoclonal anti-α-smooth muscle actin (α-SMA; Sigma, St. Louis, MO), monoclonal anti-ILK (Upstate), rabbit polyclonal anti-ILK (Upstate), mouse anti-VLA-5 (α_5_β_1_) integrin (Chemicon, Temecula, CA), hamster anti-β_1_ integrin (Santa Cruz Biotechnology, Santa Cruz, CA), mouse anti-glyceraldehyde 3-phosphate dehydrogenase (GAPDH; Ambion Inc., Austin, TX), goat anti-early endosome-associated protein 1 (EEA1; Santa Cruz Biotechnology), monoclonal anti-cellular fibronectin (Sigma), and monoclonal anti-phospho-myelin basic protein (MBP, HRP-conjugate; Upstate).

### Production and affinity-purification of polyclonal anti-ILK antibodies

Rabbits were injected with the intact 50 kDa recombinant human (rh) ILK protein which was expressed in *Escherichia coli* BL21-Rosetta strain (Novagen, Madison, WI) and purified in our laboratory. The antibodies were purified by affinity chromatography on a column with the antigen coupled to CNBr-activated Sepharose (Amersham Biosciences, Piscataway, NJ). Anti-ILK IgG (preparation R3B1) was used for the studies reported herein.

Specificity of affinity-purified antibodies was determined by immunoblot (as detailed below) against 500 ng of rhILK. ELISAs were also performed with rhILK. Briefly, graduated concentrations of rhILK were incubated in 96-well ELISA plates overnight at 4 °C. Plates were washed with buffer (Hanks Buffered Salt Solution [HBSS], 0.05% Tween-20)and blocked (HBSS, 0.1% Tween-20, and 5% casein acid hydrolysate) at room temperature for 2 h. Primary antibodies (affinity-purified, commercial monoclonal, and commercial polyclonal) were incubated with rhILK at 1 mg/ml for 2 h at room temperature. After washing, 1:5000 dilution of a secondary antibody (donkey anti-rabbit-horseradish peroxidase; Jackson ImmunoResearch Laboratories,Inc., West Grove, PA) was incubated at room temperature for 1 h. Plates were washed and incubated with peroxidase substrate (3.3',5.5'-tetramethylbenzidine; Pierce, Rockford, IL). Reactions were stopped with 10% H_3_PO_4_ and absorbance was read at 450 nm minus 650 nm on a Maxisorp 96-well ELISA plate reader (Nalge Nunc, Rochester, NY). Immunoprecipitations from LEC lysates were performed (as detailed below) with affinity-purified and commercially-available polyclonal antibodies, and subsequent immunoblots were probed with monoclonal anti-ILK antibody. Antibodies were routinely used at 1 μg/ml for immunoblotting and 10 μg/ml for immunofluorescence.

### Cell culture

C57Bl6/J x 129/SVJ mouse LEC were cultured from lens epithelial explants (described previously) [15,16]. Cells were maintained in Dulbecco's modified Eagle's medium (DMEM; Invitrogen, Carlsbad, CA) supplemented with 10% fetal bovine serum (FBS; Invitrogen), 100 U/ml penicillin G, and 100 μg/ml streptomycin SO_4_ at 37 °C in a humidified atmosphere containing 5% CO_2_. For long-term culture, cells were subcultured in 10% FBS/DMEM and were subcultured at a ratio of 1:3. A human lens epithelial cell line (SRA 01/04, provided by Dr. Venkat Reddy, Kellogg Eye Center, Ann Arbor, MI) was maintained in 10% FBS/DMEM. A human retinal pigment epithelial cell line (hRPE, ARPE-19, purchased from the ATCC) was maintained in a 1:1 mixture of DMEM and Ham's 12 media containing 20% FBS, 56 mM sodium bicarbonate, and 2 mM L-glutamine.

### Immunoblotting

Cultured cells were kept either in normal growth media or in serum-free media containing 1% BSA for 48 h to induce stress. Dissected tissues or cultured cells were subsequently lysed with ILK-lysis buffer (1% NP40, 50 mM Hepes, 150 mM NaCl, 5 mM Na_3_VO_4_, 5 mM NaF, and DNAse, 400 μg/ml) containing protease inhibitors (Pierce). Proteins were subjected to SDS-PAGE on 12% acrylamide gels. All blots were probed with anti-ILK IgG, anti-GAPDH, and in the case of the cultured cells, anti-αSMA antibodies. All primary antibody incubations were followed by an appropriate horseradish peroxidase-coupled secondary antibody (Jackson ImmunoResearch Laboratories, Inc.). The complex was detected by chemiluminescence (Supersignal West Pico reagents; Pierce).

### RT-PCR

Total RNA was isolated from lens epithelia and fiber cells of either 1-month-old C57Bl6/J x 129/SVJ mice or from cultured LEC, with the RNeasy Mini Kit (Qiagen, Valencia, CA; described previously) [17]. One μg of RNA was reverse-transcribed into cDNA with the Omniscript RT Kit (Qiagen). cDNA was amplified by use of the following primer pairs: ILK, For 5'-TTT TCA CTC AGT GCC GGG AGG-3', Rev 5'-GTG CCT TGG CTT TGT CCA CAG-3'; GAPDH, For 5'-GAC CCC TTC ATT GAC CTC AAC T-3', Rev 5'-GTT TGT GAT GGG TGT GAA CCA-3'. The PCR program was 12 min at 94 °C, followed by cycles of 45 s at 95 °C, 59 s at 65 °C, and 2 min at 72 °C, followed by a final extension of 8 min at 72 °C. GAPDH was used as an internal control for normalization to a reference standard. PCR products were separated on 2% agarose gels and were visualized by staining with ethidium bromide.

### Immunohistochemistry

Eyeballs from C57Bl6/J x 129/SVJ 1-month-old mice [18] were fixed in 3.7% paraformaldehyde for 4 h at room temperature or were frozen directly at -80 °C in OCT compound (Sakura, Torrance, CA). Paraformaldehyde-fixed eyeballs were dehydrated in a series of ethanol solutions and were embedded in paraffin. Sections were subsequently deparaffinized and rehydrated. Antigen unmasking was performed with Auto/Zyme (Biomeda, Foster City, CA). Frozen eyeballs were sectioned on a cryostat at 6-8 μ. These frozen sections were then warmed to room temperature, fixed in 4 °C acetone, and allowed to air-dry. Nonspecific binding sites of all sections were blocked with 20% Aquablock (East Coast Biologics Inc., North Berwick, MA) in PBS-Tween (0.2%) for 1 h. Sections were subsequently incubated with R3B1 anti-ILK antibody (10 μg/ml) for 2 h at room temperature, washed in PBS-Tween, and exposed to goat anti-rabbit IgG conjugated to FITC (Molecular Probes) for 1 h. All staining antibodies described above were used at concentrations according to manufacturer's protocols. Cell nuclei were stained with Hoechst 33258 fluorochrome (4 μg/ml; Molecular Probes). Negative controls included replacement of primary antibody by non-immune rabbit IgG, commercial polyclonal anti-ILK (Upstate), or R3B1 preabsorbed overnight at 4 °C with 10 μg/ml rhILK.

### mRNA Silencing

To decrease ILK expression during lens epithelial outgrowth from mouse lens explants, we cultured the explants (previously described) [15,16] with the following modifications. Six μl of HiPerFect (Qiagen) was incubated with either GFP-targeting siRNA (control) or an equimolar mixture of 3 ILK-targeting siRNA constructs (#SI01076047, #SI01076054, and #SI01076061; Qiagen) in serum-free DMEM, according to the manufacturer's protocol. Two mouse lens explants for each sample were initially cultured in DMEM containing 20% FBS and either a GFP- or an ILK-targeting siRNA transfection mixture. The same siRNA mixtures were added to cultures every 72 h for the duration of the explant culture. At 7 and 14 days after the explants were started, samples were processed as described above and were analyzed by immunoblotting for EMT markers.

### Immunocytochemistry

Cells grown overnight on glass coverslips in 10% FBS were washed with PBS and were subsequently fixed with 3.7% formaldehyde in PBS. Coated coverslips were incubated overnight at 4 °C with 10 μg/ml human plasma fibronectin (Calbiochem, La Jolla, CA). Staining was performed with affinity-purified anti-ILK IgG ± an anti-α_5_β_1_ integrin mouse IgG (10 μg/ml) or anti-β_1_ integrin hamster IgG (10 μg/ml), followed by a fluorescein isothiocyanate or tetramethylrhodamine isothiocyanate (FITC/TRITC)-conjugated goat anti-rabbit IgG, goat anti-mouse IgG, or goat anti-hamster IgG, respectively (7.5 μg/ml; Jackson ImmunoResearch Laboratories). Nuclei were stained with Hoechst dye 33258 (4 μg/ml; Molecular Probes, Eugene, OR) for 2 min. Cells were photographed with a Leica fluorescence microscope (Leica Microsystems AG Wetzlar, Germany).

### ILK immunoprecipitation and in vitro activity assay

ILK activity was determined as previously described, with minor modifications [8]. After treatment, cells were lysed in ILK lysis buffer. Lysates (250 μg) were incubated with 2 μg of one of three products: commercial or affinity-purified rabbit anti-ILK polyclonal IgG (R3B1), non-specific rabbit IgG, or beads alone, for 16 h at 4 °C with gentle agitation. Immune complexes were purified through incubation with Protein A/G-Sepharose (Santa Cruz Biotechnology) for 1 h at 4 °C, followed by two sequential washes in ILK lysis buffer and two sequential washes in ILK kinase buffer (50 mM HEPES, 10 mM MgCl_2_, 2 mM MnCl_2_, 5 mM Na_3_VO_4_, and 5 mM NaF). Standard immunoprecipitation reactions were halted after 2 washes with ILK lysis buffer and were resolved by SDS-PAGE. For ILK activity assays, the final wash was removed, and bead-protein complexes were subsequently incubated with kinase buffer, containing 200 mM ATP and 5 μg of MBP, at 30 °C for 25 min. Reactions were quenched with 2x SDS-PAGE sample buffer containing 1 mM dithiothreitol (DTT) and were heated at 90 °C for 10 min. Samples were resolved by SDS-PAGE, transferred onto polyvinylidene difluoride-plus membranes (Millipore, Billerica, MA), and immunoblotted with horseradish peroxidase-conjugated mouse anti-phospho-MBP monoclonal antibody (Upstate).

## Results

### Generation of a polyclonal anti-ILK antibody

**Figure 1:**
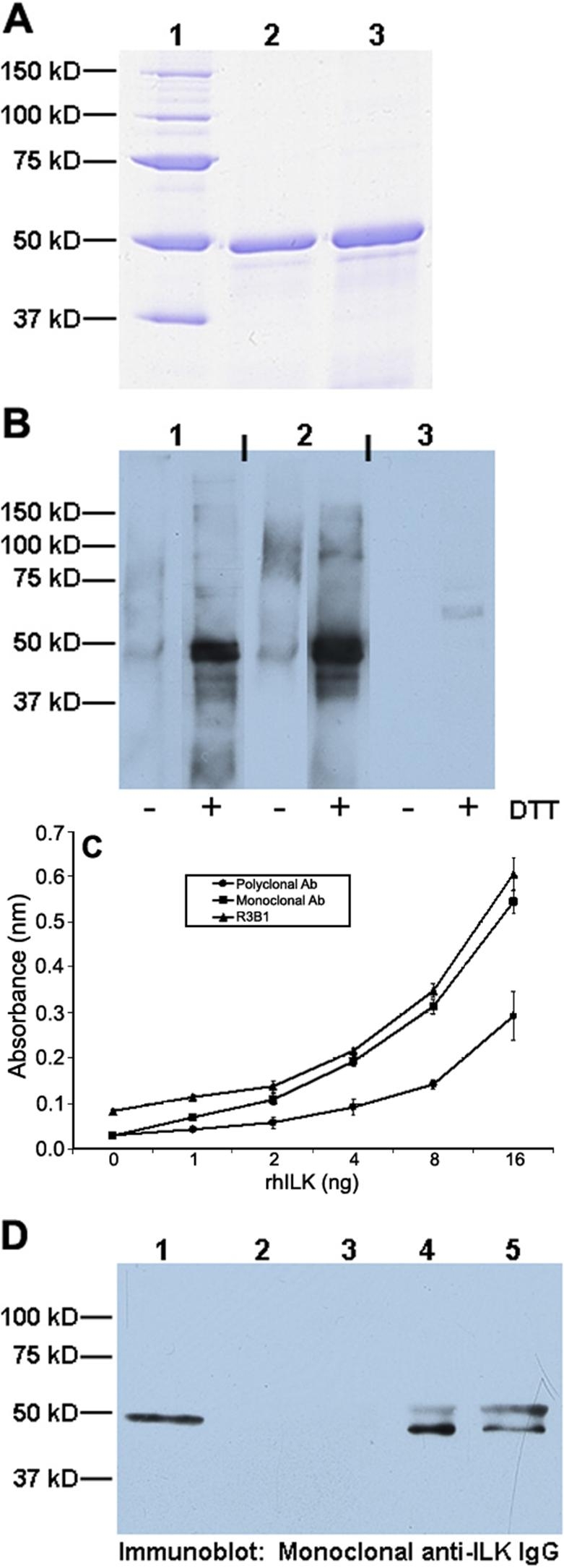
Characterization of polyclonal ILK antibody. **A**: Purified rhILK used for antigen in rabbits. Lane 1, molecular weight standards; lane 2, 5 μg rhILK; lane 3, 10 μg rhILK. **B**: Immunoblot of immortalized mouse LEC lysates probed with affinity-purified polyclonal ILK IgG (R3B1; column 1), in comparison with commercially-available anti-ILK monoclonal (column 2) and polyclonal antibodies (column 3), before (-) and after (+) reduction with dithiothreitol (DTT). **C**: ELISA showing binding of affinity-purified anti-ILK IgG (R3B1) to rhILK (triangle) in comparison with commercially-available anti-ILK polyclonal antibody (circle) and monoclonal antibodies (square). Each point represents three independent experiments, ±SD. **D**: Comparison of ILK immunoprecipitated from mouse LEC lysates with commercial and R3B1 IgG and subsequently probed with commercial monoclonal antibody. Lane 1, rhILK; lane 2, lysate only (no antibody); lane 3, non-specific rabbit IgG; lane 4, commercial polyclonal antibody; lane 5, R3B1. In **A**, **B**, and **D**, molecular weight markers are shown on the left of each panel.

rhILK (about 50 kDa) was expressed in *E. coli* and was purified for use as an antigen in rabbits (Figure 1A). Serum protein from injected rabbits was affinity-purified and used for immunoblotting against reduced and non-reduced rhILK; these results were compared directly with those obtained with commercially-available mouse monoclonal and rabbit polyclonal anti-ILK reagents (Figure 1B). Affinity-purified R3B1 exhibited specificity similar to that of the monoclonal reagent, whereas the commercial polyclonal antibody did not recognize the nonreduced substrate and identified a band of alternate size (about 60 kDa) within the reduced rhILK sample. Figure 1C shows an ELISA comparing the three antibodies against increasing concentrations of rhILK. Affinity-purified R3B1 exhibited binding comparable to that of the monoclonal reagent, while the commercial polyclonal reagent showed a significant decrease in binding to purified rhILK.

To determine whether R3B1 could immunoprecipitate ILK, we combined lysate from cultured mouse LEC with identical concentrations of either control rabbit IgG, commercial polyclonal anti-ILK IgG, or R3B1 IgG. The resulting immunoprecipitates were resolved by SDS-PAGE and were immunoblotted with the monoclonal anti-ILK antibody (Figure 1D). Both commercial and R3B1 polyclonal antibodies immunoprecipitated ILK from mouse cell lysates, without any cross-reactivity seen with either beads alone (Figure 1D; lane 2) or with nonspecific rabbit IgG (Figure 1D; lane 3). Therefore, affinity-purified R3B1 showed strong reactivity with rhILK, comparable to that of the commercial monoclonal anti-ILK antibody. In contrast, the commercial polyclonal antibody recognized a protein of different mass on immunoblots only under reducing conditions and displayed less recognition of antigen by ELISA (Figure 1C). Both commercial and R3B1 polyclonal antibodies immunoprecipitated ILK from mouse cell lysates (Figure 1D).

### ILK expression in the mouse lens

**Figure 2:**
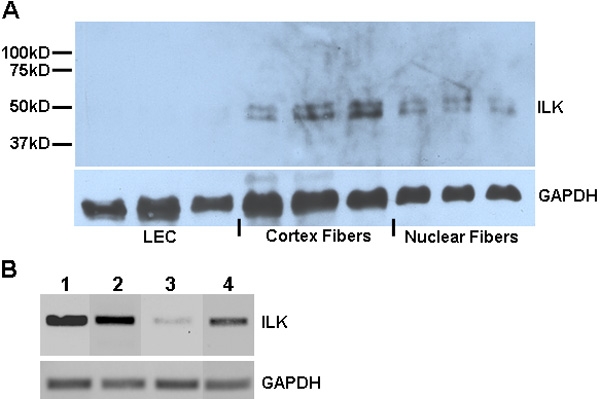
ILK in the mouse lens and in immortalized lens cells. **A**: Western blot of lysates from cortical and nuclear fiber cells dissected from mouse lenses. Additional probe for GAPDH is shown as a protein loading control. Molecular weight markers are indicated on the left. **B**: RT-PCR for ILK performed on RNA isolated from immortalized mouse (lane 1) and human LEC (lane 2), as well as mouse lens tissue dissected into capsule/epithelial (lane 3) and lens fiber fractions (lane 4). Probe for GAPDH mRNA is shown as a control for cellular RNA.

For evaluation of ILK expression in the mouse lens, 1-month-old eyes were dissected into the following fractions: capsule/LEC, cortical fiber cells, and nuclear fiber cells, the lysates of which were used for immunoblotting. Three separate samples were probed with R3B1 for the presence of ILK (Figure 2A). Relative to LEC, ILK protein was increased in both cortical and nuclear fiber cell fractions. RT-PCR was also performed on RNA isolated from dissected lenses (LEC and fiber fractions) and two immortalized LEC lines, in which one was derived from a mouse and the other from a human (Figure 2B). LEC derived from lens explants showed significantly less ILK mRNA than either the fiber cell fraction or the immortalized human and mouse LEC lines.

**Figure 3:**
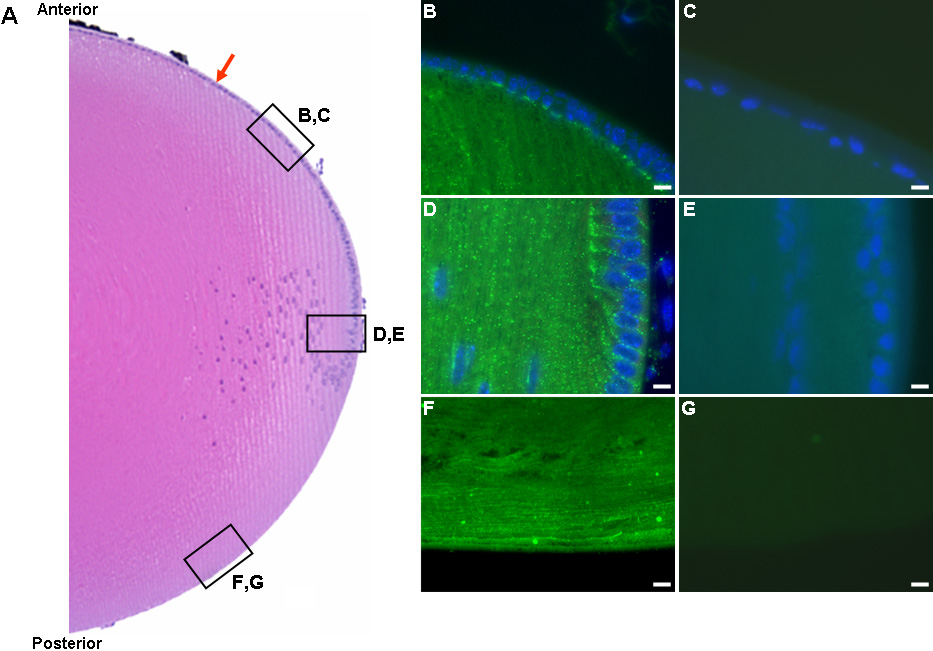
Localization of ILK in the mouse lens. **A**: Hematoxylin and eosin-stained mouse lens. The red arrow denotes the lens epithelial cell layer. Boxes indicate anterior (**B**,**C**), equatorial (**D**,**E**), and posterior (**F**,**G**) areas imaged for immunohistochemistry. **B**: Immunohistochemistry of mouse lens anterior LEC and fiber cells with affinity-purified anti-ILK IgG (R3B1). FITC-conjugated donkey anti-rabbit IgG was used as a secondary antibody. **C**: Immunohistochemistry of anterior LEC with commercial polyclonal anti-ILK antibody. **D**: Immunohistochemistry of lens equatorial region with R3B1. **E**: Immunohistochemistry of lens equatorial region with R3B1 pre-absorbed with 10 μg/ml rhILK. **F**: Immunohistochemistry of the posterior lens with R3B1. **G**: Immunohistochemistry of the posterior lens with rabbit IgG control. The scale bar is equal to 10 μm.

To define further the expression pattern of ILK in the lens, we embedded whole lenses for sectioning and immunofluorescence (Figure 3). A non-specific isotype control IgG, the commercial rabbit polyclonal anti-ILK IgG, or R3B1 anti-ILK antibodies preabsorbed with rhILK demonstrated the specificity of the R3B1 antibody (Figure 3C,E,G, respectively; see Figure 3A for reference areas shown in Figure 3B-G). Staining of the LEC along the anterior of the lens was minimal and appeared to be concentrated at the border of the LEC and newly-differentiated fiber cells (Figure 3B, bright green color). In contrast, significant staining for ILK was observed in the differentiating LEC and fiber cells of the equatorial region (Figure 3D, bright green color). Newly-differentiated fiber cells were also reactive with anti-ILK IgG along the posterior edge of the lens (Figure 3F, bright green color). The immunohistochemistry in Figure 3 revealed strong reactivity for ILK at the equatorial region and the anterior and posterior ends of newly-differentiated fiber cells, in comparison to isotype, commercial, and preabsorbed staining controls (Figure 3B-G). These data are consistent with the detection of ILK mRNA and protein in the cortical fibers but not in the majority of LEC, as shown in Figure 2.

**Figure 4:**
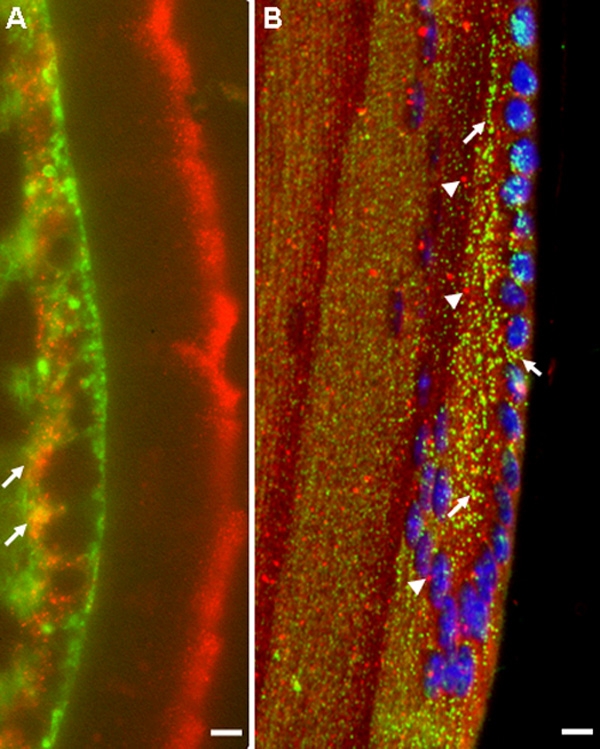
Coincidence of ILK with β_1_-integrin but not EEA1 in the mouse lens. **A**: Immunohistochemistry with R3B1 (red) and anti-β_1_ integrin antibody (green). Yellow indicates coincidence of staining (arrows). The scale bar is equal to 1 μm. **B**: Immunohistochemistry with R3B1 (green; arrows) and anti-EEA1 antibody (red; arrowheads). No coincidental staining is evident. The scale bar is equal to 10 μm. Morphological alterations and non-specific staining for ILK in the capsule (**A**, extreme right) occurred with the staining protocol required for integrin immunohistochemistry.

To verify the relative cellular location of ILK in the lens, we performed simultaneous staining of ILK with either anti-β_1_-integrin or anti-EEA-1 IgG (Figure 4A,B). Staining for ILK coincided with staining for β_1_-integrin, i.e., along the LEC-fiber cell interface at the equatorial region (Figure 4A). However, there was no apparent coincidence between ILK and the endosomal marker EEA1 (Figure 4B).

### Expression of ILK as a function of EMT of LEC in vitro

**Figure 5:**
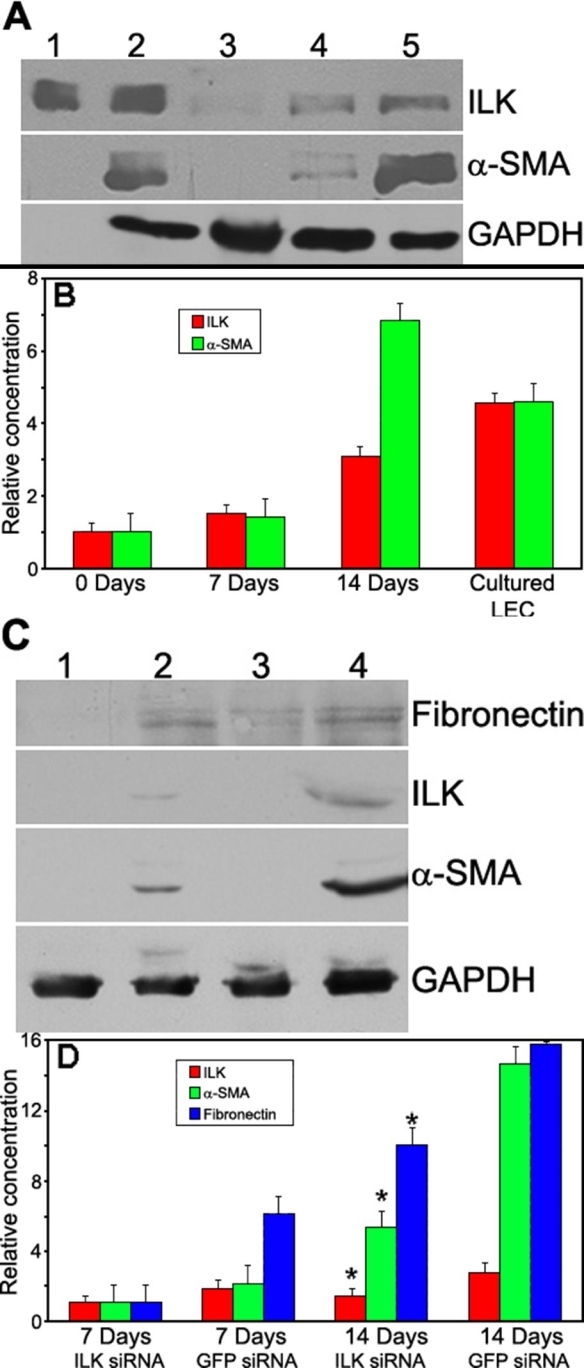
ILK is induced during LEC differentiation and inhibition of ILK retards expression of EMT markers α-SMA and fibronectin. **A**: Immunoblot of lysates (20 μg/lane) from immortalized and primary mouse LEC that grew out from dissected lens capsules (0-14 days). Lane 1, rhILK (500 ng); lane 2, immortalized LEC; lane 3, primary LEC, 0 days; lane 4, primary LEC, 7 days; lane 5, primary LEC, 14 days. Lysates were probed for ILK and α-SMA as an indicator of EMT. rhILK (lane 1) was used for protein size comparison, and a probe for GAPDH was used to normalize protein loading. **B**: Densitometry of protein bands from the blot shown in **A**. Relative expression values are normalized to those of GAPDH. **C**: Immunoblot of lysates (2 capsules/lane) from primary mouse LEC that grew out from dissected lens capsules (0-14 days). Lane 1, primary LEC + ILK-targeting siRNA, 7 days; lane 2, primary LEC + ILK-targeting siRNA, 14 days; lane 3, primary LEC + GFP-targeting siRNA (control), 7 days; lane 4, primary LEC + GFP-targeting siRNA. Lysates were probed for ILK to verify ILK-targeting siRNA efficiency. Fibronectin and α-SMA were monitored as indicators of EMT. An additional probe for GAPDH was used to normalize protein loading. **D**: Densitometry of protein bands from the blot shown in **C**. ILK- and GFP-siRNA-treated explants show a significant difference in protein expression after 14 days of culture (the asterisk indicates a p<0.02). Relative values are normalized to that of GAPDH.

As described above, the epithelial layer of the lens does not produce high levels of ILK protein in vivo until differentiation occurs. Alternatively, immortalized lens cell lines did produce ILK in vitro (Figure 2B). To resolve this apparent discrepancy, we grew primary cultures from explanted mouse lenses in culture for various times and subsequently immunoblotted lysates for ILK (Figure 5A; ILK) or α-SMA, a marker for EMT in LEC (SMA; Figure 5A). As the cells adapted to culture conditions over time, indicated by increased α-SMA production, their levels of ILK increased significantly. Within 14 days of culture, expression of α-SMA and ILK in primary LEC became comparable to that seen in the immortalized LEC lines (Figure 5A, compare lanes 2 and 5). Quantification of protein production during culture adaptation is shown in (Figure 5B). To determine whether expression of ILK was required for the increase in α-SMA seen in LEC after 14 days of culture, we cultured lens explants in the presence of control (GFP-) or ILK-targeting siRNA (Figure 5C). In addition to the decreased levels of ILK in the explanted cultures upon exposure to ILK-targeting siRNA, there was concomitantly a decreased expression of the EMT protein markers, α-SMA and fibronectin, in comparison with control samples (Figure 5C, compare lanes 2 and 4). During extended culture, inhibition of ILK did not stop the progression of EMT in explant outgrowths (as measured by augmented levels of α-SMA and fibronectin), but it did significantly retard the induction of these two markers (Figure 5D). Therefore, ILK protein expression increased upon initiation of the EMT process in vitro. When ILK was inhibited by siRNA, the EMT progression slowed significantly, the data indicating a role for ILK in EMT of mouse LEC.

### ILK expression in immortalized mouse lens epithelial cells

**Figure 6:**
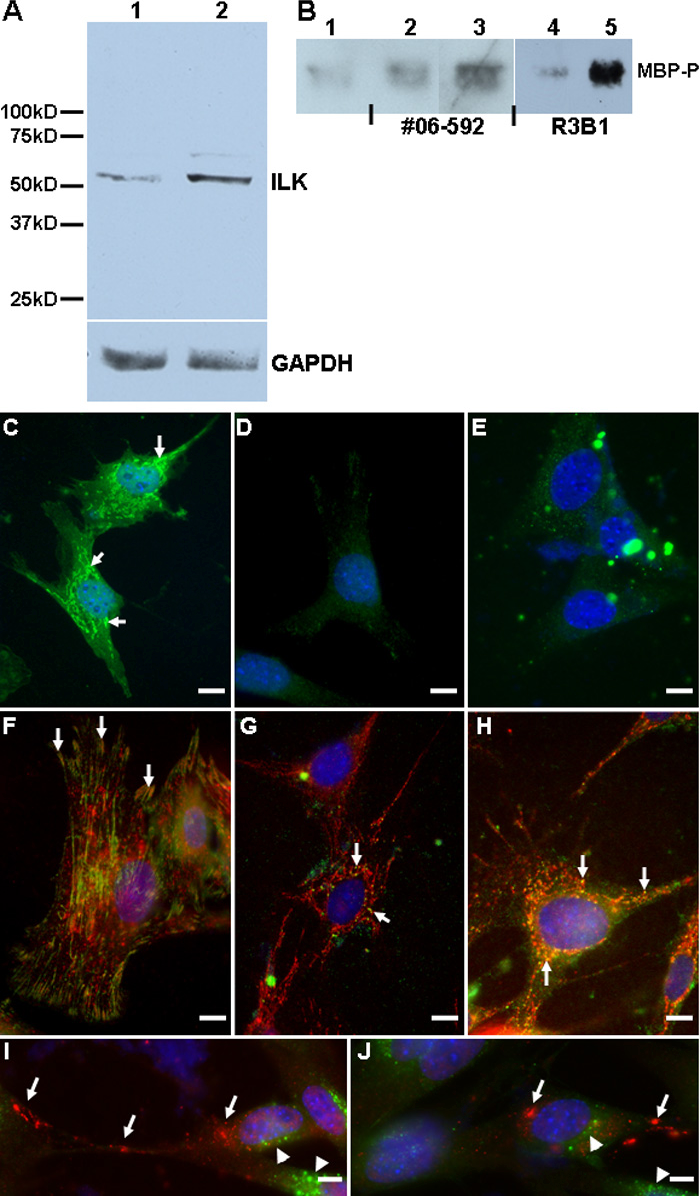
Production and localization of ILK in cultured mouse LEC. **A**: Immunoblot of lysates (20 μg/lane) from resting (lane 1) and serum-deprived cultured LEC (lane 2). Additional probe for GAPDH is shown as a protein loading control. Molecular weight standards are indicated at left. **B**: Immunoblot of phosphorylated MBP (MBP-P) following immunoprecipitation of ILK with commercial (#06-592) or R3B1 polyclonal antibodies, followed by in vitro kinase assay. **C**: Immunocytochemistry with affinity-purified anti-ILK IgG (R3B1) of LEC plated on glass coverslips; arrows indicate ILK. FITC-conjugated donkey anti-rabbit IgG was used as a secondary antibody. **D**: Staining performed with rabbit IgG control. **E**: Staining with R3B1 antibody preincubated with 10 mu g/ml rhILK. **F**: Staining with R3B1 (red) and anti-β_1_ integrin antibody (green). Yellow indicates coincidence of staining (arrows). **G**: Staining of cells with R3B1 (red) and anti-α5β_1_ integrin antibody (green). **H**: Staining with R3B1 (red) and anti-α5β_1_ integrin antibody (green) of cells plated on glass coverslips coated with 10 μg/ml fibronectin. Arrows in **G** and **H** indicate coincidence of staining. **I**-**J**: Staining with R3B1 (red) and anti-EEA1 antibody (green). Arrows indicate ILK; arrowheads indicate EEA1. The scale bar is equal to 1 μm.

To examine ILK expression in cultured mouse LEC, we probed lysates of resting and serum-depleted cells with R3B1 (Figure 6A). ILK was expressed in cultured LEC and its expression increased under the stress of serum-deprivation (Figure 6A, compare lanes 1 and 2). In vitro kinase assays were also performed with the commercial anti-ILK and the affinity-purified R3B1 polyclonal antibodies, both of which immunoprecipitated ILK from resting and serum-deprived LEC. Purified MBP, an ILK substrate, was added to the reaction after precipitation and the phosphorylated form was probed after SDS-PAGE (Figure 6B). Commercial and R3B1 polyclonal antibodies precipitated active ILK in the kinase assay. Serum-deprivation of cells resulted in increased phosphorylated MBP (MBP-P) relative to resting cells and isotype immunoprecipitation controls.

**Figure 7:**
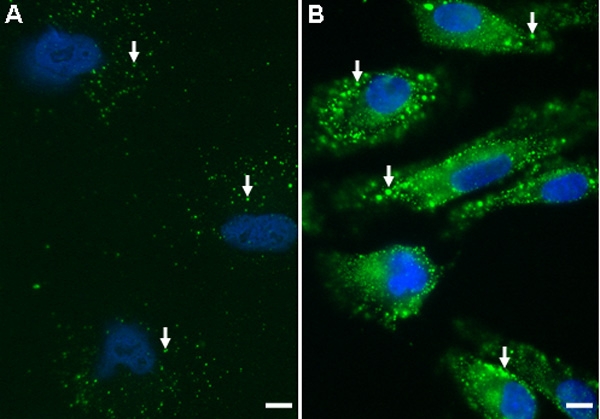
Localization and increased production of ILK in cultured human RPE. Immunocytochemistry with affinity-purified anti-ILK IgG (R3B1) of hRPE plated on glass coverslips. FITC-conjugated donkey anti-rabbit IgG was used as a secondary antibody. **A**: Non-stressed hRPE stained with R3B1 (green). **B**: hRPE deprived of serum for 24 h and subsequently stained with R3B1 (green). Increased ILK expression was apparent after stress. Arrows indicate localized ILK staining in hRPE. The scale bar is equal to 1 μm.

Immunocytochemistry with R3B1 was performed on LEC plated on glass coverslips (Figure 6C-J). Staining of ILK was clearly evident in cultured LEC (Figure 6C) relative to the IgG control (Figure 6D) and could be competed by prior saturation of the antibody with rhILK (Figure 6E). Pre-absorption of R3B1 with rhILK resulted in some fluorescent precipitate that appeared across the field in a plane above the cells (aggregates in Figure 6E). Colocalization of ILK with β_1_-integrin was also evident in cultured LEC, with specific staining apparent along the edges of cells (Figure 6F, arrows). Colocalization of ILK with the fibronectin-binding integrin α_5_β_1_ was also investigated on non-coated (Figure 5G) and fibronectin-coated coverslips (Figure 6H). Coating with fibronectin increased both the apparent levels of α_5_β_1_ integrin and its colocalization with ILK (arrows, Figure 6H). The distribution of ILK in cultured LEC did not coincide with that of the endosomal marker EEA1 (Figure 6I,J), a result consistent with data from sectioned lenses (Figure 4B). Staining by R3B1 IgG was also present in resting and stressed hRPE plated on glass coverslips (Figure 7).

LEC, therefore, produced ILK, seen as a specific band (about 50 kDa) in immunoblotted lysates. An increase in ILK protein synthesis was apparent in cells following stress. Both commercial and R3B1 polyclonal antibodies were effective in the immunoprecipitation of active ILK for in vitro kinase assays. Staining of cultured cells showed strong reactivity and specificity for ILK, whereas incubation with rhILK significantly decreased intracellular staining. ILK, identified by R3B1, colocalized with both β_1_-integrin and α_5_β_1_ integrin, which was increased after the plating of cells on fibronectin-coated coverslips. Importantly, ILK did not co-localize with the endosomal marker EEA1. Moreover, the distribution of ILK in LEC appeared unique in comparison with that of another immortalized, ocular epithelial cell line, hRPE.

## Discussion

ILK functions in several diverse biological processes including cellular proliferation, survival, motility, and differentiation. In animal models ILK has been shown to have important activities in tumor growth/progression, angiogenesis, and tissue differentiation. However, little is known about the function of ILK in the mammalian eye, specifically in the lens. The specialized functions required of the lens epithelial layer for lens maturation and continued transparency make subsets of these cells prime candidates for the expression of ILK. In addition, LEC plays an important role in the potential development of PCO after cataract surgery, a process entailing EMT [19]. For these reasons we explored the expression and role of ILK in the mouse lens and during EMT of lens cells in vitro. It was first necessary, however, to produce and characterize a new anti-ILK antibody.

Previous studies on ILK have been performed with commercially-available reagents that have varied reactivity against ILK and identify proteins with different molecular masses (or possibly, different proteins). Production of an affinity-purified, polyclonal antibody against rhILK (R3B1) proved an effective solution to the variability of available reagents. R3B1 IgG performed reproducibly for immunoblotting, immunoprecipitation, and immunohisto/cytochemistry under different protocols of fixation and staining. The purified antibody recognized a band on SDS-PAGE of 50 kDa after reduction and was reactive in ELISA with sensitivity similar to that of the available monoclonal reagent. Staining with the antibody showed specific reactivity in tissue and cells that could be competed by pre-incubation of the antibody with rhILK. Results from immunoblotting, immunocytochemistry, and immunohistochemistry with R3B1 have been comparable to those with the monoclonal antibody [5,20,21]. However, the monoclonal antibody has not been verified for immunoprecipitation of ILK. We therefore demonstrate the purification of a polyclonal anti-ILK antibody with the specificity and reactivity of a commercially-available monoclonal antibody. The polyclonal R3B1 was effective in immunoprecipitation, immunostaining, and in vitro kinase assays.

While the presence of ILK in the mouse lens was expected, expression was surprisingly localized to the differentiating LEC of the equatorial region and to fiber cells, with little to no expression in the anterior LEC layer. These data were confirmed by immunoblot of dissected lens fractions. Results from RT-PCR were also consistent with data derived from immunoblots, which illustrated the low levels of ILK mRNA present in isolated LEC that increased after differentiation of LEC into fiber cells. Levels of ILK were also substantial in two immortalized lens cell lines.

Immunohistochemistry was subsequently performed on the lens to verify specific localization of ILK in differentiating LEC and fiber cells. Staining revealed a concentration of ILK along the interface marking the posterior edge of the equatorial LEC and anterior ends of newly-differentiated fiber cells. Staining for ILK continued posterior to the equatorial region along the lateral membranes of the most recently-differentiated fiber cells. Significant levels of ILK were also observed at the lens anterior surface, apparently localized along fiber cell membranes. Immunostaining for ILK and β_1_-integrin showed coincidence at the LEC-fiber cell interface, anterior to the equatorial region of the lens. The localization of ILK deposits in the lens coincides with immunohistochemistry performed in the developing murine brain that revealed foci of ILK at the interface between differentiating tissue layers in a membrane-associated pattern [5].

ILK, with its known association with the cytoplasmic tails of integrin β_1_- and β_3_-subunits, would be expected to function optimally at integrin-localized areas. Previous work has shown a distribution of β_1_-integrin, unique among tissue epithelia, at both basal and apical membranes of LEC and differentiating fiber cells [22,23]. Of specific interest are the alterations in integrins required during the differentiation of LEC and subsequent fiber cell elongation, migration, and survival. Fiber cell maturation requires migration along the anterior of the lens where little to no ECM is deposited. Additionally, these cells must survive a modified apoptotic process resulting in denucleation, elimination of most organelles, and significant cytoskeletal alterations (reviewed in [24]). The interface between LEC and fiber cells has been shown to be a site of changing integrin interactions and expression by newly-differentiated fiber cells (reviewed in [25]). Of note are alterations observed in the α_6_-integrin subunit, a binding partner of β_1_-integrin. α_6_-integrin expression, cytoskeletal interaction, and anti-apoptotic signaling are upregulated in LEC as they enter the equatorial region, and these changes continue during fiber cell differentiation, even in a microenvironment (the LEC-fiber cell interface) which exhibits a paucity of ECM deposition [26-29]. Specifically in the lens fiber cells, α_6_-integrin has been shown to stimulate PI3K-dependent signaling, which regulates cytoskeletal reorganization and survival during the process of denucleation [29]. Downstream of PI3K/AKT signaling, inhibition of GSK-3β has been postulated to act as a survival mechanism during lens differentiation [29]. In addition to the PI3K pathway, GSK-3b can be directly inhibited by ILK phosphorylation in a PI3K-dependent manner (reviewed in [1]). As ILK has been previously shown to be involved in cell survival and cytoskeletal alteration, we postulate that the unexpected distribution of ILK in the lens is due in part to the lens-specific expression pattern of its known interacting integrin partners. This localization would allow ILK to function as a positive regulator of cytoskeletal alteration, migration, and survival in LEC and fiber cells during differentiation in the lens.

Despite little to no protein detected by either immunoblotting or immunohistochemistry, the dissected LEC fraction contained ILK mRNA. It is possible that contamination of LEC or fiber cells during dissection, or the inclusion of cells from the equatorial region, could account for the faint ILK mRNA signal in the LEC fraction. Whereas ILK expression by LEC in vivo appears minimal, it is upregulated during their differentiation into fiber cells. As ILK has been implicated in EMT [12-14] and is expressed by most cultured cells (reviewed in [1]), we proceeded to determine the expression pattern of ILK and its potential role during culture of explanted LEC.

The process of EMT in LEC has been seen during the adaptation of lens cells to culture conditions and as a result of cataract surgery, as LEC escaping surgical removal regrow on the posterior surface of the lens implant. These changes include the increased production of survival factors and changes in cytoskeletal and integrin proteins [11]. We investigated the role of ILK in this adaptation process by characterizing immortalized LEC and primary LEC outgrowths from lens explants. Over a 2 week interval, immunoblotting and staining showed strong ILK expression in the cell lines and increased levels of ILK in primary mouse LEC. The production of ILK by the explanted LEC was increased concomitantly with that of the EMT markers, α-SMA and fibronectin. Similar results were obtained with regard to ILK and the EMT process in renal tubular epithelial cells [12,14]. ILK-targeting siRNAs were next used to inhibit ILK expression during EMT of the explant cultures. siRNA transfection eliminated the majority of ILK expression (about 70%) over the explant culture period (14 days). This inhibition of ILK production was associated with a retardation of EMT progression, as indicated by the reduced levels of α-SMA and fibronectin. These results substantiate the proposed role for ILK as a positive regulator during LEC EMT and as a target for retardation of PCO following cataract surgery [11].

In immortalized LEC, ILK could also be induced by stress in the form of serum deprivation. The removal of serum led to an increase in protein expression and ILK kinase activity, as verified by phosphorylation of its target MBP. ILK was localized in cultured LEC in a distinctive pattern resembling that of certain cytoplasmic proteins including tubulin, lens-specific microtubules, and some of the actin-associated proteins. Although increases in ILK protein were evident in both LEC and cultured hRPE after stress (Figure 6A and Figure 7), a similar pattern of ILK localization was not observed in cultured hRPE (compare Figure 6C and Figure 7). It is, therefore, possible that EMT-mediated localization of ILK occurs in cultured LEC. Enhanced production of specific EMT-induced cytoskeletal proteins could potentiate ILK binding and ILK-induced motility, as has been previously reported for cultured myocytes [21]. Work is ongoing to identify EMT-associated and/or lens-specific cytoskeletal components that could account for the staining pattern observed for ILK in cultured LEC. As expected, coincident staining of ILK and β_1_ integrin, as well as the α_5_β_1_ complex, was observed in cultured LEC. Moreover, colocalization was enhanced after α_5_β_1_ integrin was activated by its preferred ECM ligand, fibronectin.

The use of a novel anti-ILK antibody has allowed us to identify ILK in the newly-differentiated fiber cells of the mouse lens. Although there do not appear to be substantial amounts of ILK mRNA or protein in the anterior lens epithelia, there is an increase in expression as LEC enter the equatorial region of the lens and begin differentiation into fiber cells, a result consistent with recently published data [30]. In addition, ILK production in LEC significantly increased as these cells were grown in vitro, data lending credence to the hypothesis that ILK plays a role in LEC EMT and potentially in the PCO process that can occur after cataract surgery.

## References

[1] Attwell S Mills J Troussard A Wu C Dedhar S Integration of cell attachment, cytoskeletal localization, and signaling by integrin-linked kinase (ILK), CH-ILKBP, and the tumor suppressor PTEN. Mol Biol Cell 2003 14 4813 25 12960424 10.1091/mbc.E03-05-0308 PMC284786

[2] Liu E Sinha S Williams C Cyrille M Heller E Snapper SB Georgopoulos K St-Arnaud R Force T Dedhar S Gerszten RE Targeted deletion of integrin-linked kinase reveals a role in T-cell chemotaxis and survival. Mol Cell Biol 2005 25 11145 55 16314534 10.1128/MCB.25.24.11145-11155.2005 PMC1316981

[3] Lee SP Youn SW Cho HJ Li L Kim TY Yook HS Chung JW Hur J Yoon CH Park KW Oh BH Park YB Kim HS Integrin-linked kinase, a hypoxia-responsive molecule, controls postnatal vasculogenesis by recruitment of endothelial progenitor cells to ischemic tissue. Circulation 2006 114 150 9 16818815 10.1161/CIRCULATIONAHA.105.595918

[4] Ishii T Satoh E Nishimura M Integrin-linked kinase controls neurite outgrowth in N1E-115 neuroblastoma cells. J Biol Chem 2001 276 42994 3003 11560928 10.1074/jbc.M105198200

[5] Belvindrah R Nalbant P Ding S Wu C Bokoch GM Muller U Integrin-linked kinase regulates Bergmann glial differentiation during cerebellar development. Mol Cell Neurosci 2006 33 109 25 16914328 10.1016/j.mcn.2006.06.013

[6] Friedrich EB Clever YP Wassmann S Werner N Bohm M Nickenig G Role of integrin-linked kinase in vascular smooth muscle cells: regulation by statins and angiotensin II. Biochem Biophys Res Commun 2006 349 883 9 16962068 10.1016/j.bbrc.2006.07.217

[7] Mills J Niewmierzycka A Oloumi A Rico B St-Arnaud R Mackenzie IR Mawji NM Wilson J Reichardt LF Dedhar S Critical role of integrin-linked kinase in granule cell precursor proliferation and cerebellar development. J Neurosci 2006 26 830 40 16421303 10.1523/JNEUROSCI.1852-05.2006 PMC2757417

[8] Barker TH Baneyx G Cardo-Vila M Workman GA Weaver M Menon PM Dedhar S Rempel SA Arap W Pasqualini R Vogel V Sage EH SPARC regulates extracellular matrix organization through its modulation of integrin-linked kinase activity. J Biol Chem 2005 280 36483 93 16115889 10.1074/jbc.M504663200

[9] Boulter E Grall D Cagnol S Van Obberghen-Schilling E Regulation of cell-matrix adhesion dynamics and Rac-1 by integrin linked kinase. FASEB J 2006 20 1489 91 16723384 10.1096/fj.05-4579fje

[10] Hannigan G Troussard AA Dedhar S Integrin-linked kinase: a cancer therapeutic target unique among its ILK. Nat Rev Cancer 2005 5 51 63 15630415 10.1038/nrc1524

[11] de Iongh RU Wederell E Lovicu FJ McAvoy JW Transforming growth factor-beta-induced epithelial-mesenchymal transition in the lens: a model for cataract formation. Cells Tissues Organs 2005 179 43 55 15942192 10.1159/000084508

[12] Li Y Yang J Dai C Wu C Liu Y Role for integrin-linked kinase in mediating tubular epithelial to mesenchymal transition and renal interstitial fibrogenesis. J Clin Invest 2003 112 503 16 Erratum in: J Clin Invest. 2004; 113:491 12925691 10.1172/JCI17913 PMC171389

[13] Ahmed N Maines-Bandiera S Quinn MA Unger WG Dedhar S Auersperg N Molecular pathways regulating EGF-induced epithelio-mesenchymal transition in human ovarian surface epithelium. Am J Physiol Cell Physiol 2006 290 C1532 42 16394028 10.1152/ajpcell.00478.2005

[14] Shimizu M Kondo S Urushihara M Takamatsu M Kanemoto K Nagata M Kagami S Role of integrin-linked kinase in epithelial-mesenchymal transition in crescent formation of experimental glomerulonephritis. Nephrol Dial Transplant 2006 21 2380 90 16728424 10.1093/ndt/gfl243

[15] Yan Q Weaver M Perdue N Sage EH Matricellular protein SPARC is translocated to the nuclei of immortalized murine lens epithelial cells. J Cell Physiol 2005 203 286 94 15534859 10.1002/jcp.20226

[16] Weaver MS Sage EH Yan Q Absence of SPARC in lens epithelial cells results in altered adhesion and extracellular matrix production in vitro. J Cell Biochem 2006 97 423 32 16211577 10.1002/jcb.20654

[17] Yan Q Blake D Clark JI Sage EH Expression of the matricellular protein SPARC in murine lens: SPARC is necessary for the structural integrity of the capsular basement membrane. J Histochem Cytochem 2003 51 503 11 12642629 10.1177/002215540305100412

[18] Perdue N Yan Q Caveolin-1 is up-regulated in transdifferentiated lens epithelial cells but minimal in normal human and murine lenses. Exp Eye Res 2006 83 1154 61 16914142 10.1016/j.exer.2006.06.007

[19] Awasthi N Wagner BJ Suppression of human lens epithelial cell proliferation by proteasome inhibition, a potential defense against posterior capsular opacification. Invest Ophthalmol Vis Sci 2006 47 4482 9 17003443 10.1167/iovs.06-0139

[20] Li F Zhang Y Wu C Integrin-linked kinase is localized to cell-matrix focal adhesions but not cell-cell adhesion sites and the focal adhesion localization of integrin-linked kinase is regulated by the PINCH-binding ANK repeats. J Cell Sci 1999 112 4589 99 10574708 10.1242/jcs.112.24.4589

[21] Chen H Huang XN Yan W Chen K Guo L Tummalapali L Dedhar S St-Arnaud R Wu C Sepulveda JL Role of the integrin-linked kinase/PINCH1/alpha-parvin complex in cardiac myocyte hypertrophy. Lab Invest 2005 85 1342 56 16170337 10.1038/labinvest.3700345

[22] Bassnett S Missey H Vucemilo I Molecular architecture of the lens fiber cell basal membrane complex. J Cell Sci 1999 112 2155 65 10362545 10.1242/jcs.112.13.2155

[23] Menko AS Philip NJ Beta 1 integrins in epithelial tissues: a unique distribution in the lens. Exp Cell Res 1995 218 516 21 7540985 10.1006/excr.1995.1186

[24] Yan Q Liu JP Li DW Apoptosis in lens development and pathology. Differentiation 2006 74 195 211 16759286 10.1111/j.1432-0436.2006.00068.x

[25] Sue Menko A Lens epithelial cell differentiation. Exp Eye Res 2002 75 485 90 12457861 10.1006/exer.2002.2057

[26] Walker JL Menko AS alpha6 Integrin is regulated with lens cell differentiation by linkage to the cytoskeleton and isoform switching. Dev Biol 1999 210 497 511 10357906 10.1006/dbio.1999.9277

[27] Walker JL Zhang L Menko AS A signaling role for the uncleaved form of alpha 6 integrin in differentiating lens fiber cells. Dev Biol 2002 251 195 205 12435352 10.1006/dbio.2002.0823

[28] Walker JL Zhang L Zhou J Woolkalis MJ Menko AS Role for alpha 6 integrin during lens development: Evidence for signaling through IGF-1R and ERK. Dev Dyn 2002 223 273 84 11836791 10.1002/dvdy.10050

[29] Weber GF Menko AS Phosphatidylinositol 3-kinase is necessary for lens fiber cell differentiation and survival. Invest Ophthalmol Vis Sci 2006 47 4490 9 17003444 10.1167/iovs.06-0401

[30] Wederell ED de Iongh RU Extracellular matrix and integrin signaling in lens development and cataract. Semin Cell Dev Biol 2006 17 759 76 17134921 10.1016/j.semcdb.2006.10.006

